# Supplementary material for: An O2-sensing stressosome from a Gram-negative bacterium
Source: Nat Commun. 2016 Aug 4;7:12381. doi: 10.1038/ncomms12381 (PMC4976288; doi:10.1038/ncomms12381)
Supplement: Supplementary Information — Supplementary Figures 1-15, Supplementary Tables 1-4 and Supplementary References [file ncomms12381-s1.pdf]

An O_2-Sensing Stressosome from a Gram Negative Bacterium

Xin Jia, Jian-bo Wang, Shannon Rivera, Duc Duong, and Emily E. Weinert

Supplementary Information

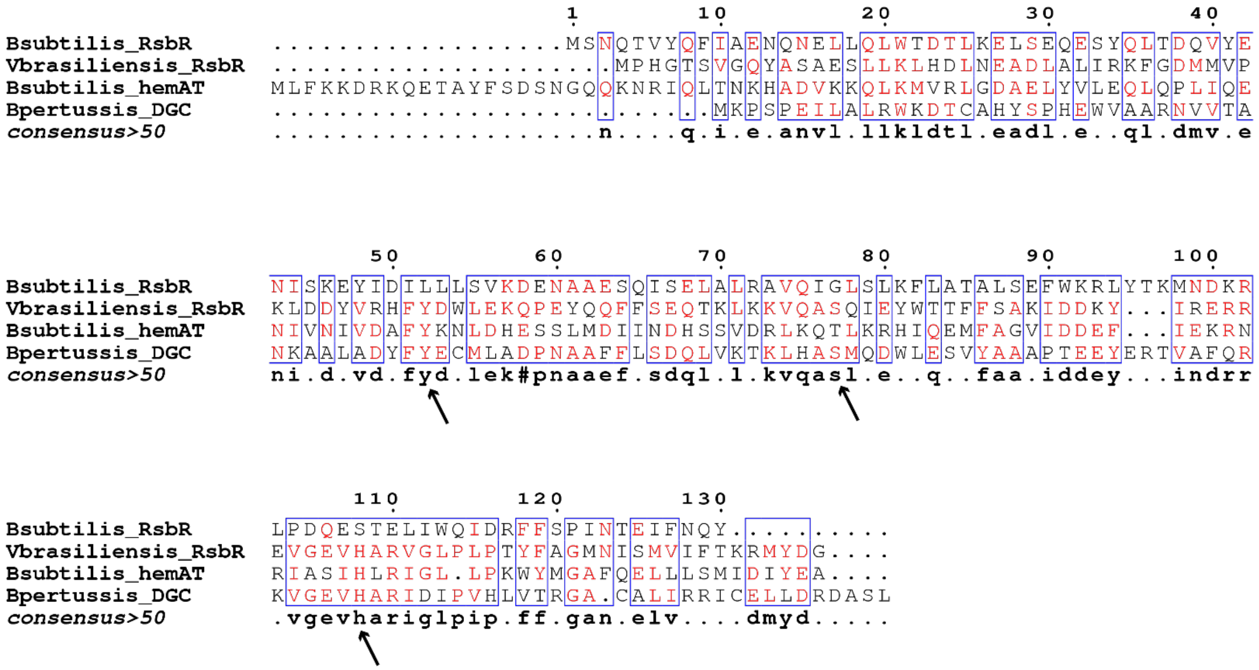

Supplementary Figure 1. Sequence alignment of N-terminal globin domain of *V. brasiliensis* RsbR (Vbrasiliensis_RsbR; WP_006879723), non-heme globin domain of *B. subtilis* RsbR (Bsubtilis_RsbR; WP_009966610), and sensor globin domains from globin coupled sensors in *B. subtilis* (HemAT; WP_015252424) and *Bordetella pertussis* (*Bpe*GReg; WP_050861487). Arrows point to the proximal histidine and distal tyrosine and serine residues required for heme and ligand binding, and are conserved in sensor globin domains (Bsubtilis_hemAT, Bpertussis_DGC, *V. brasiliensis*_RsbR) but not in *B. subtilis* non-heme globin domain (Bsubtilis_RsbR).

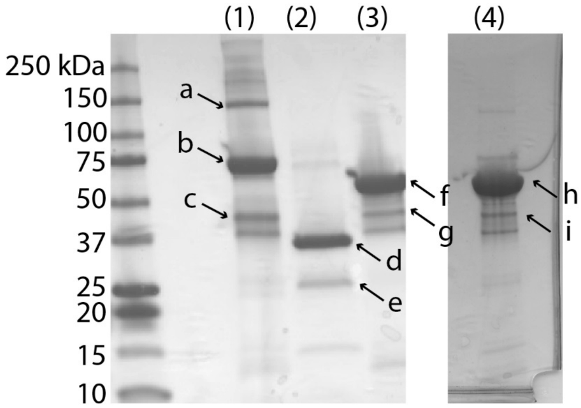

Supplementary Figure 2. SDS-PAGE analysis of purified stressosome components RsbR, RsbS and RsbT from *V. brasiliensis*, and mutant RsbT D78N, stained with Coomasie. His_6-MBP-RsbR (Lane 1, b, 75 kDa, 5 μM), GST-RsbS (Lane 2, d, 39 kDa, 5 μM), His_6-MBP-RsbT (Lane 3, f, 58 kDa, 5 μM) and mutant RsbT (Lane 4, h, D87N, 10 μM) were separated on 4-20% acrylamide gels. N-terminal His_6-MBP or GST tagged constructs of RsbR, RsbS, and RsbT were used to improve stability of the proteins during purification and analysis. Bands at 42.5 (Lane 1, c; lane 3, g and lane 4 i) and 26 kDa (Lane 2, e) are small amount of detached MBP or GST tags. Higher molecular band at 150 kDa (Lane 1, a) corresponds to RsbR dimer that was not fully denatured.

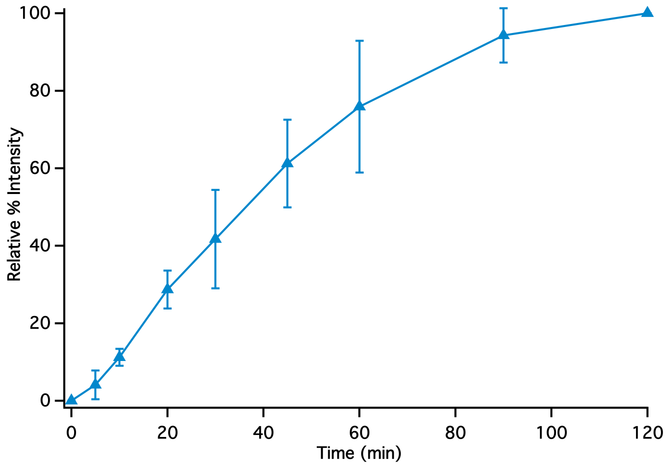

Supplementary Figure 3. Average autophosphorylation kinetics of kinase RsbT. Kinetic experiments were run in triplicate, and mean and standard deviation values were calculated for each time point. Rate of autophosphorylation = 0.015 ± 0.002 min^{-1}.

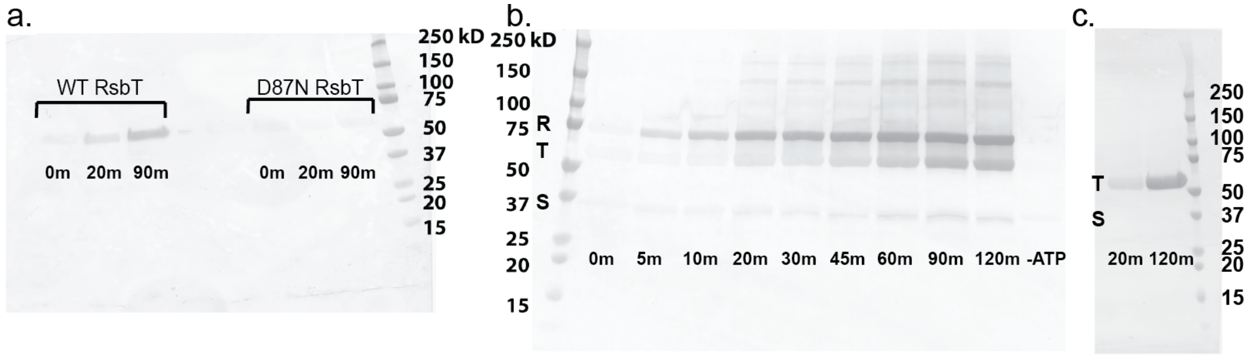

Supplementary Figure 4. Representative western blots probing kinase activities. **(a)** Comparison of RsbT (Lane (1)-(3)) and mutant RsbT D87N (Lane (4)-(6)) autophosphorylation activity. RsbT autophosphorylation reactions were quenched at 0 min, 20 min, and 90 min. RsbTD87N autophosphorylation reactions were quenched at 0 min, 20 min, and 90 min. **(b)** RsbT kinase activity in the presence of RsbS and RsbR Fe^{II} state. Reactions were quenched at 0 min, 5 min, 10 min, 20 min, 30 min, 45 min, 60 min, 90 min, 120 min. Negative control reaction (**-ATP**) was performed without addition of ATPγS to ensure there was no cross-activity with alkylated cysteine. **(c)** Phosphorylation reaction containing RsbS and RsbT. RsbT autophosphorylation is clearly visible; however, phosphorylation of RsbS is not detected.

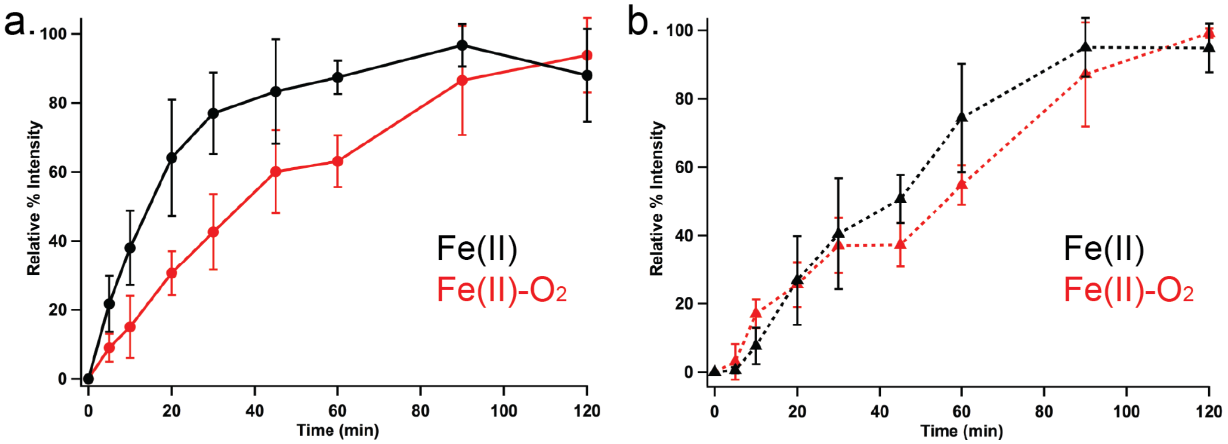

Supplementary Figure 5. Average phosphorylation kinetics of kinase RsbT in a RsbR/T mixture. Average phosphorylation kinetics of RsbR (**A**) and RsbT (**B**). Kinetic experiments were run in at least triplicate, and mean and standard deviation values were calculated for each time point. Rate of RsbR phosphorylation for Fe^{II} = 0.057 ± 0.005 min^{-1}; Fe^{II}-O_2 = 0.016 ± 0.001 min^{-1}.

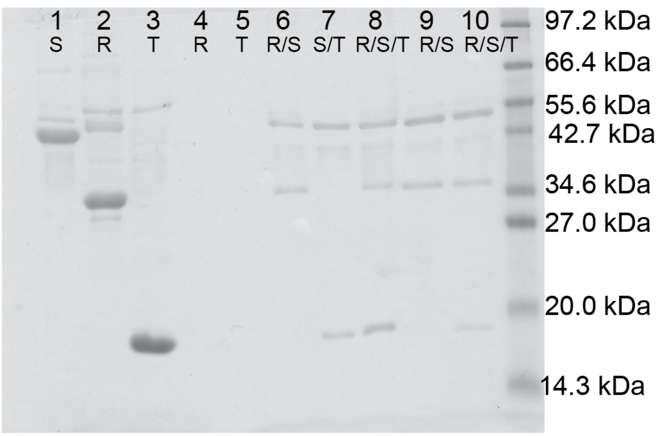

Supplementary Figure 6. Representative pull-down assay. Lane 1, His-MBP-RsbS; lane 2, RsbR; lane 3, RsbT; lane 4, pull down of RsbR; lane 5, pull-down of RsbT; lane 6, Fe^{II}-O_2 RsbR+His-MBP-RsbS; lane 7, His-MBP-RsbS+RsbT; lane 8, Fe^{II}-O_2 RsbR+His-MBP-RsbS+RsbT; lane 9, Fe^{II} RsbR+His-MBP-RsbS; lane 10, Fe^{II} RsbR+His-MBP-RsbS+RsbT; marker lane. Lanes 1, 2, and 3 were not subjected to Ni-NTA pull down and were included as standards for facile identification of the various proteins. Lanes 4 and 5 are controls to determine non-specific interactions between RsbR (lane 4) and RsbT (lane 5) with the Ni-NTA resin.

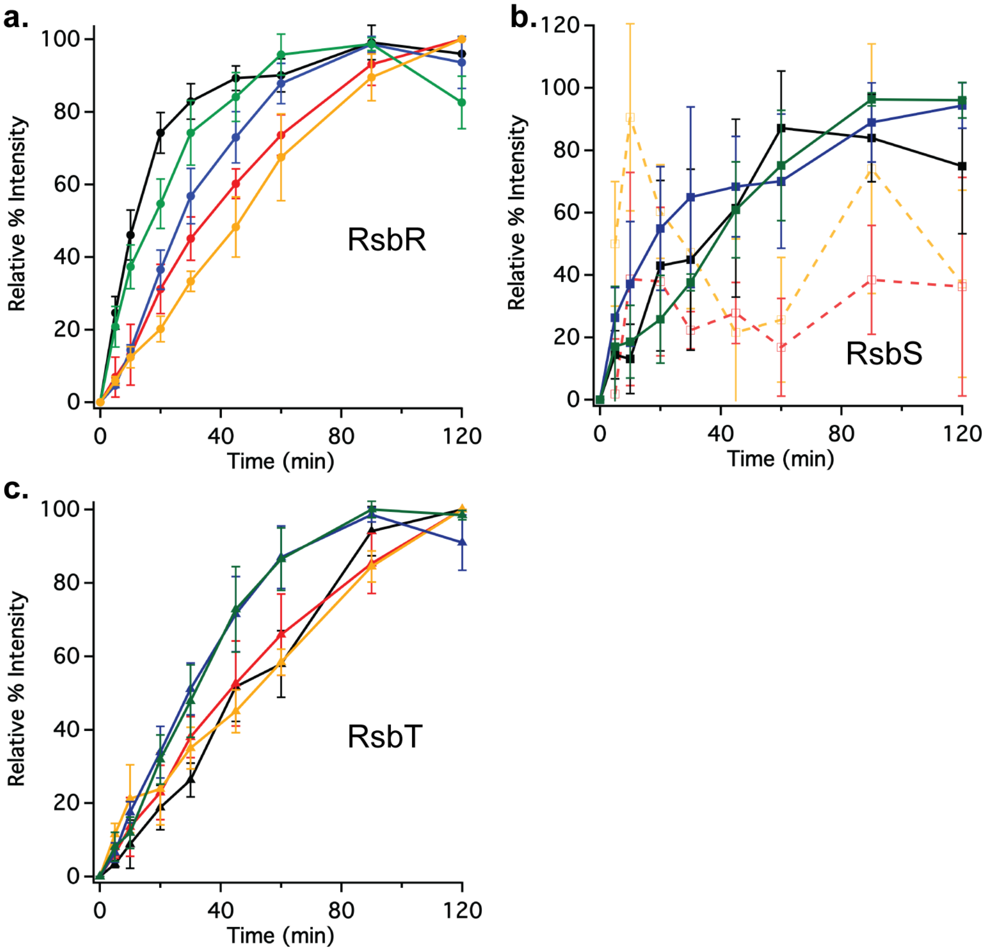

Supplementary Figure 7. Phosphorylation kinetics of kinase RsbT in the presence of RsbR in various ligation states and RsbS. Average rate of phosphorylation of RsbR (**a**), RsbS (**b**) and RsbT (**c**) in a RsbR/S/T mixture (ligation/oxidation states of RsbR: Fe^{III}, yellow; Fe^{II}-O_2, red; Fe^{II}-CO, blue; Fe^{II}-NO, green; Fe^{II} unligated, black). All experiments were performed at least in triplicate, and mean and standard deviation values are plotted for each time point. Phosphorylation of RsbS did not increase above background levels when Fe^{III} or Fe^{II}-O_2 RsbR was included in the reaction mixture (variability in the RsbS Fe^{II}-O_2 and Fe^{III} plots is due to normalization within the background signals).

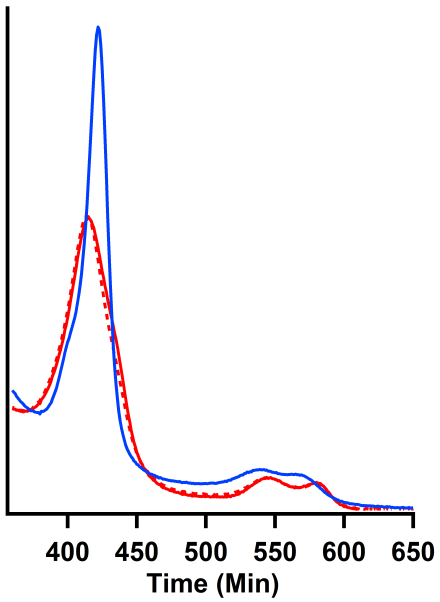

Supplementary Figure 8. Autooxidation UV-Vis spectra of *V. brasiliensis* RsbR in RsbR/S/T complex. Spectra of RsbR Fe^{II}-O_2 in RsbR/S/T were taken at immediately after mixing with oxygenated buffer (solid red line; 416, 544, 580 nm) and after two hours (dashed red line; 417, 544, 580 nm). Heme ligand CO for Fe^{II} RsbR was added after two hours to produce diagnostic RsbR Fe^{II}-CO spectra (blue; 421, 540, 570 nm).

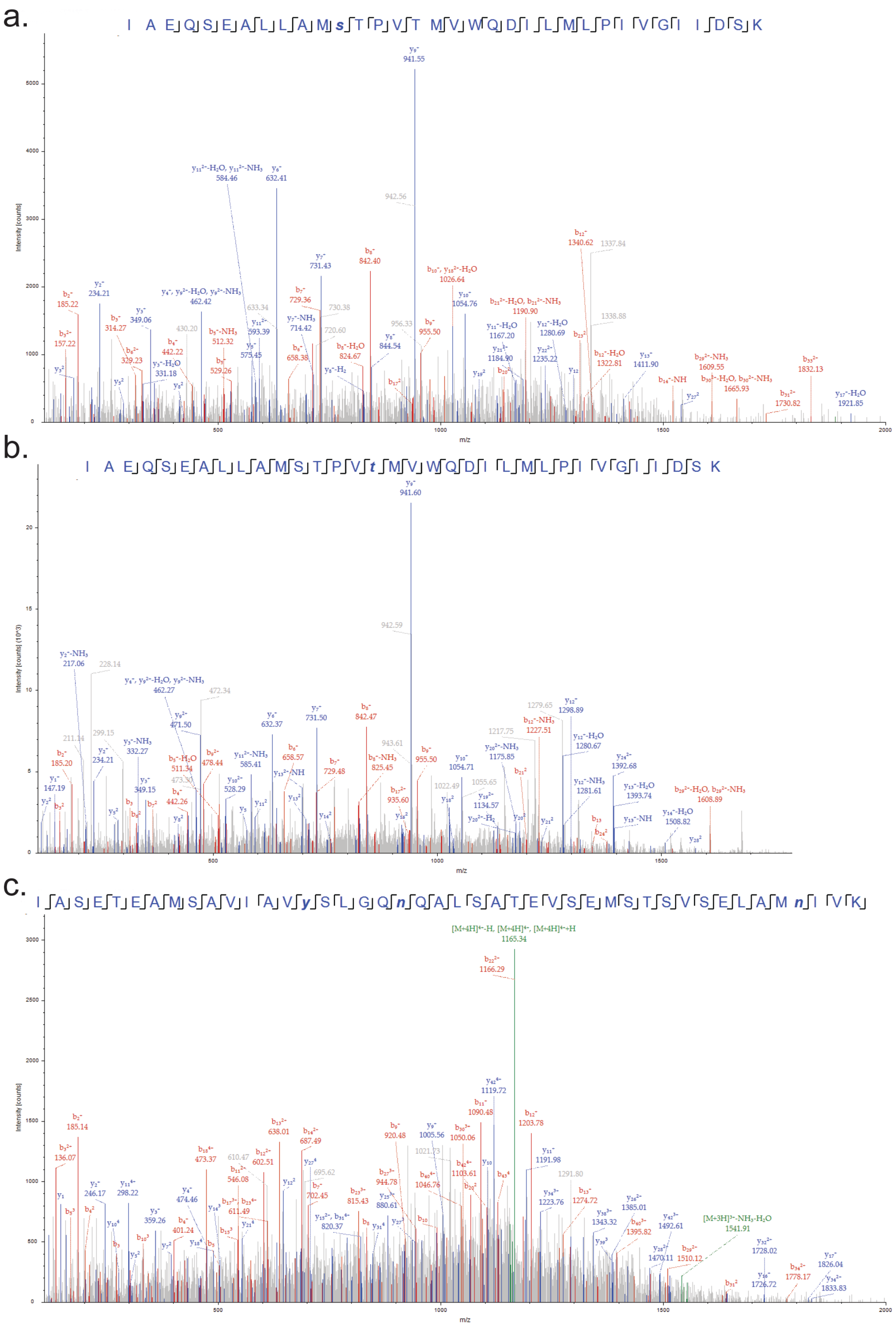
a.
b.
c.
Intensity [counts]
Intensity [counts] (10^3)
m/z
y9+
941.55
y9+
941.60
[M+4H]4+-H, [M+4H]4+, [M+4H]4++H
1165.34

d.

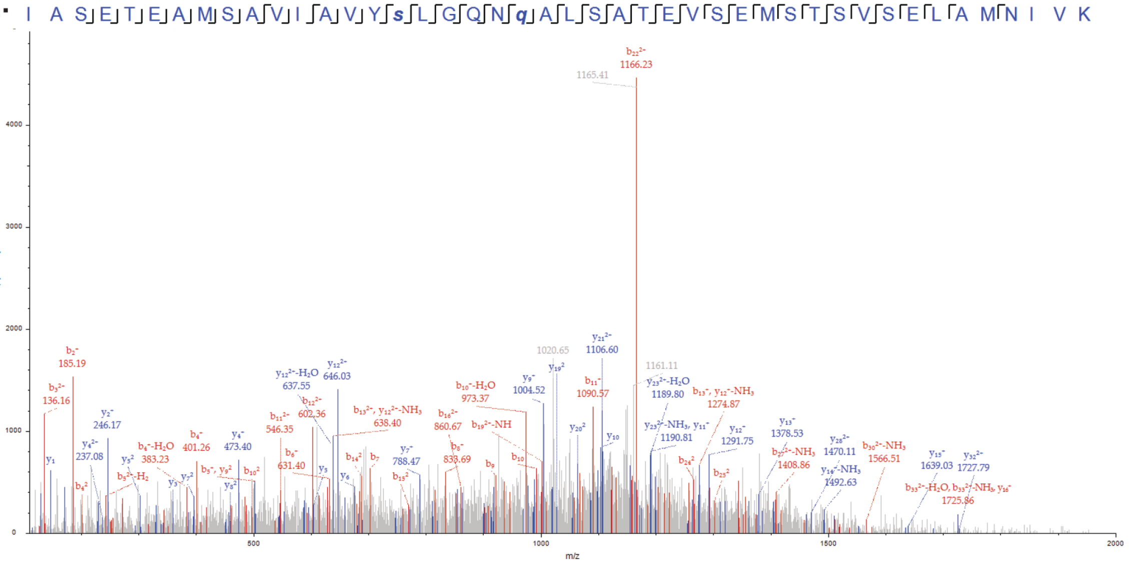

e.

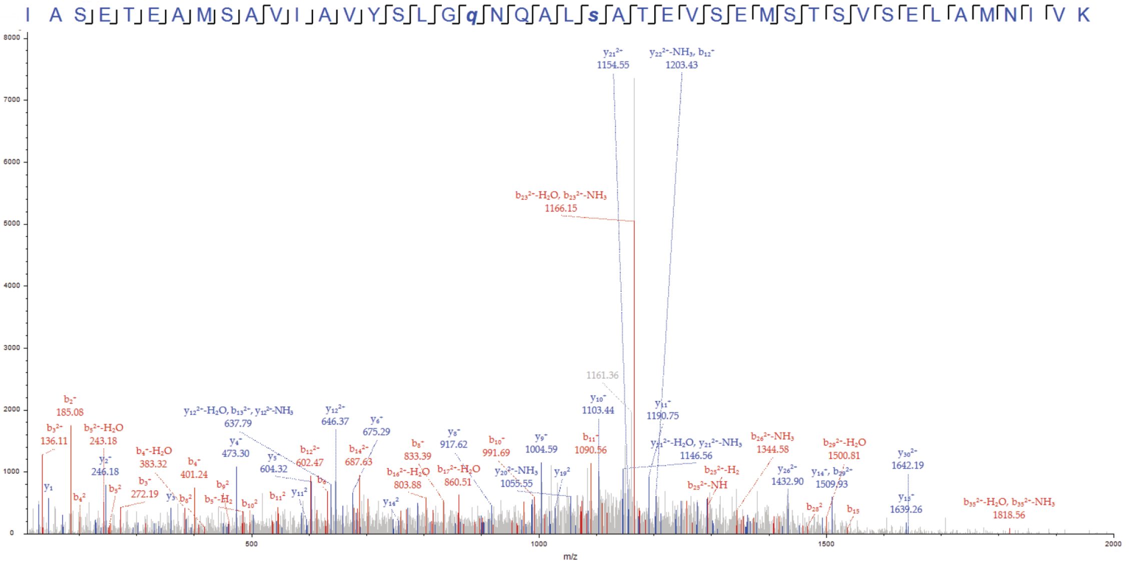

Supplementary Figure 9. Mass spectra with fragmentation of RsbR and RsbT thiophosphorylated peptides. Thiophosphorylated sites are marked as bold italicized lowercase letters.. **(a)** RsbR S178 **(b)** RsbR T182 **(c)** RsbT Y30 **(d)** RsbT S31 **(e)** RsbT S39.

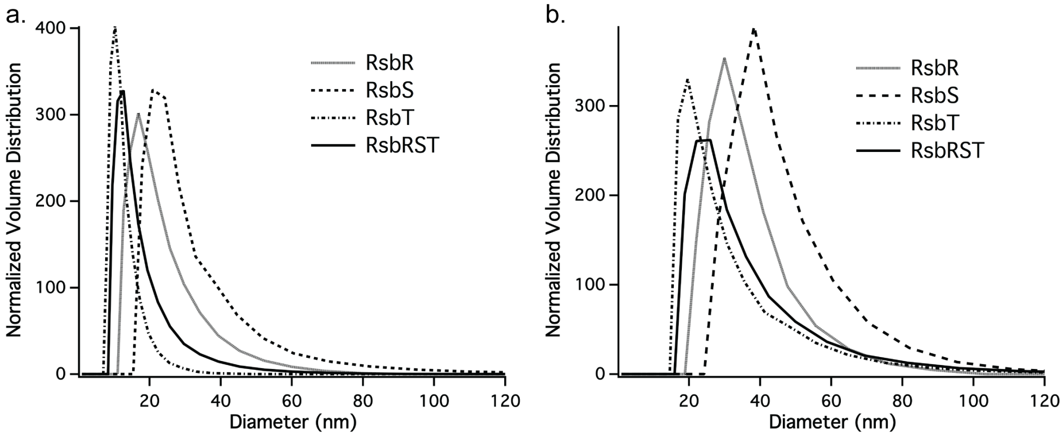

Supplementary Figure 10. (**a**) Dynamic light scattering (DLS) of tagged RsbR (15.1 nm), RsbS (20.5 nm), RsbT (9.8 nm) and the RsbR/S/T mixture (15.8 nm) when RsbR is in Fe^{II}-O_2 ligation state; (b) DLS of tag-cleaved RsbR (34 nm), RsbS (43.3 nm), RsbT (27.3 nm), and RsbR/S/T mixture (30.2 nm) when RsbR is in Fe^{II}-O_2 ligation state. Data was fit using the Marquadt function.

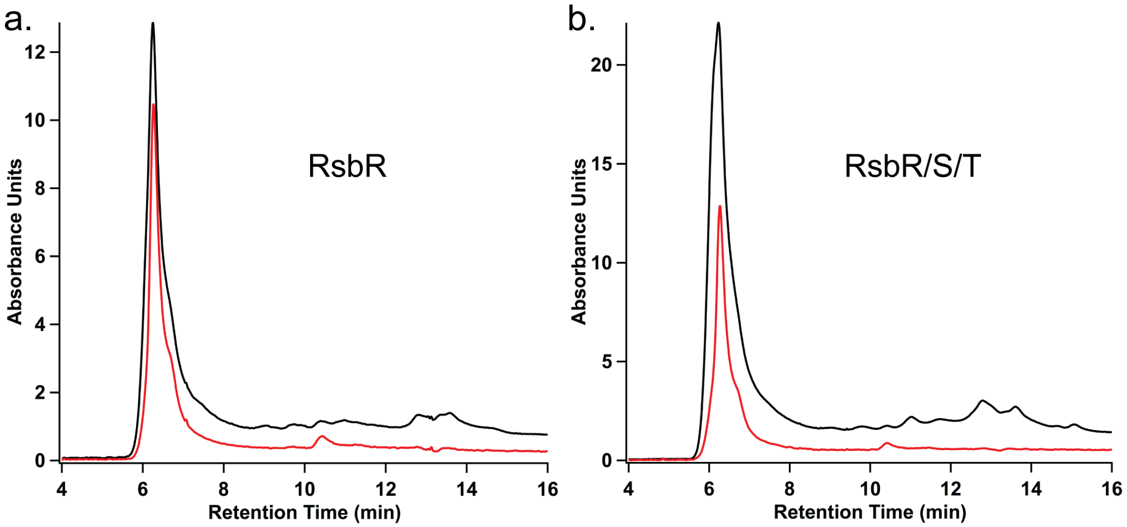

Supplementary Figure 11. Analytical gel filtration analysis of RsbR and RsbR/S/T. Red, 416 nm (heme absorbance maximum); black, 280 nm (protein absorbance). Individual proteins and protein mixtures form large complexes in solution. The ratio of heme absorbance to protein absorbance decreases in the RsbR/S/T mixture due to the three proteins interacting and co-eluting (RsbS and RsbT do not contribute to the heme absorbance, only the absorbance at 280 nm).

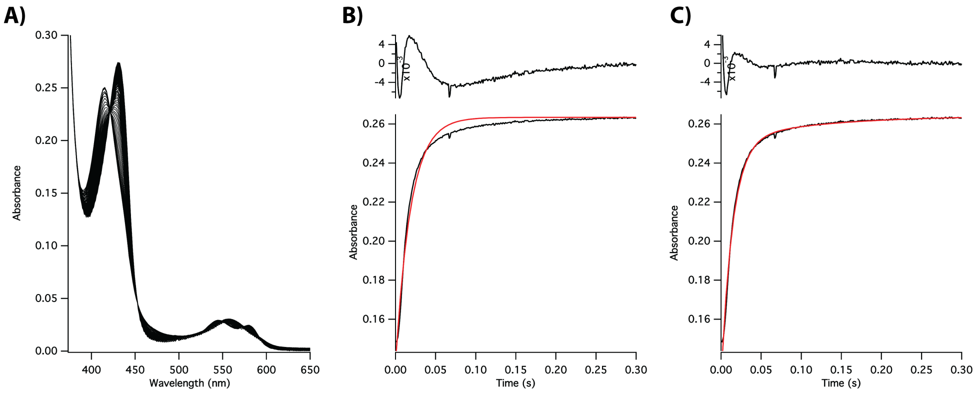

Supplementary Figure 12. Stopped flow kinetics. (**A**) Representative overlayed stopped flow spectra of purified RsbR from *V. Brasilienesis.* Comparison of mono-exponential and bi-exponential fits (**B** and **C**, respectively) for representative O_2 dissociation experiments for RsbR. Raw data at 434 nm is shown in black and the calculated fit curve is in red. The fit residuals (difference between raw and fitted data) are shown in a black curve above each plot.

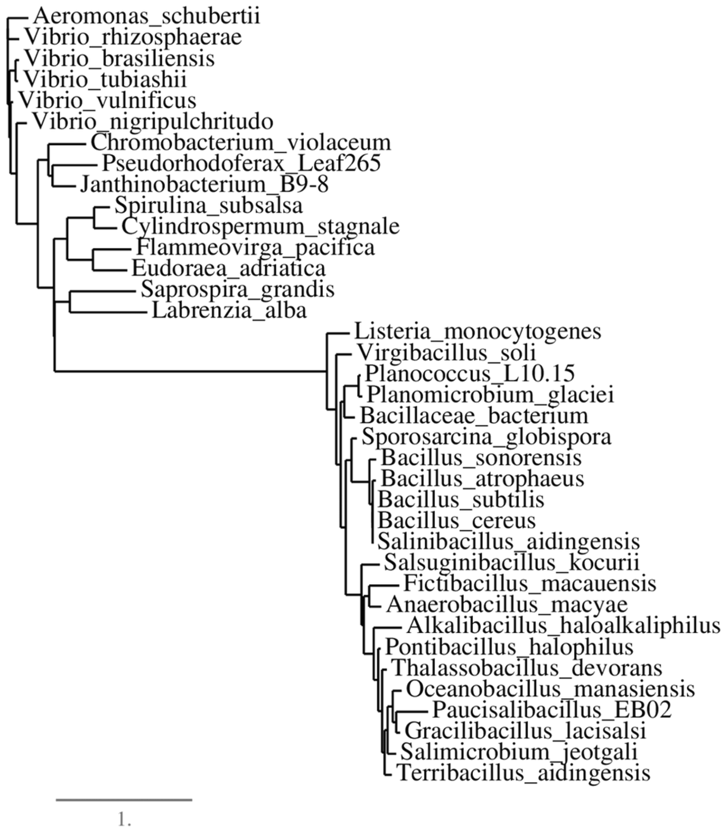

Supplementary Figure 13. Phylogenetic analysis of sensor globin-containing and non-heme globin-containing RsbR proteins. RsbR proteins from *Vibrio* species and *Bacillus* species cluster into two separate clades (branch support value = 1; branch length is proportional to number of substitutions per site; bootstrap = 250). Image produced using Phylogeny [1].

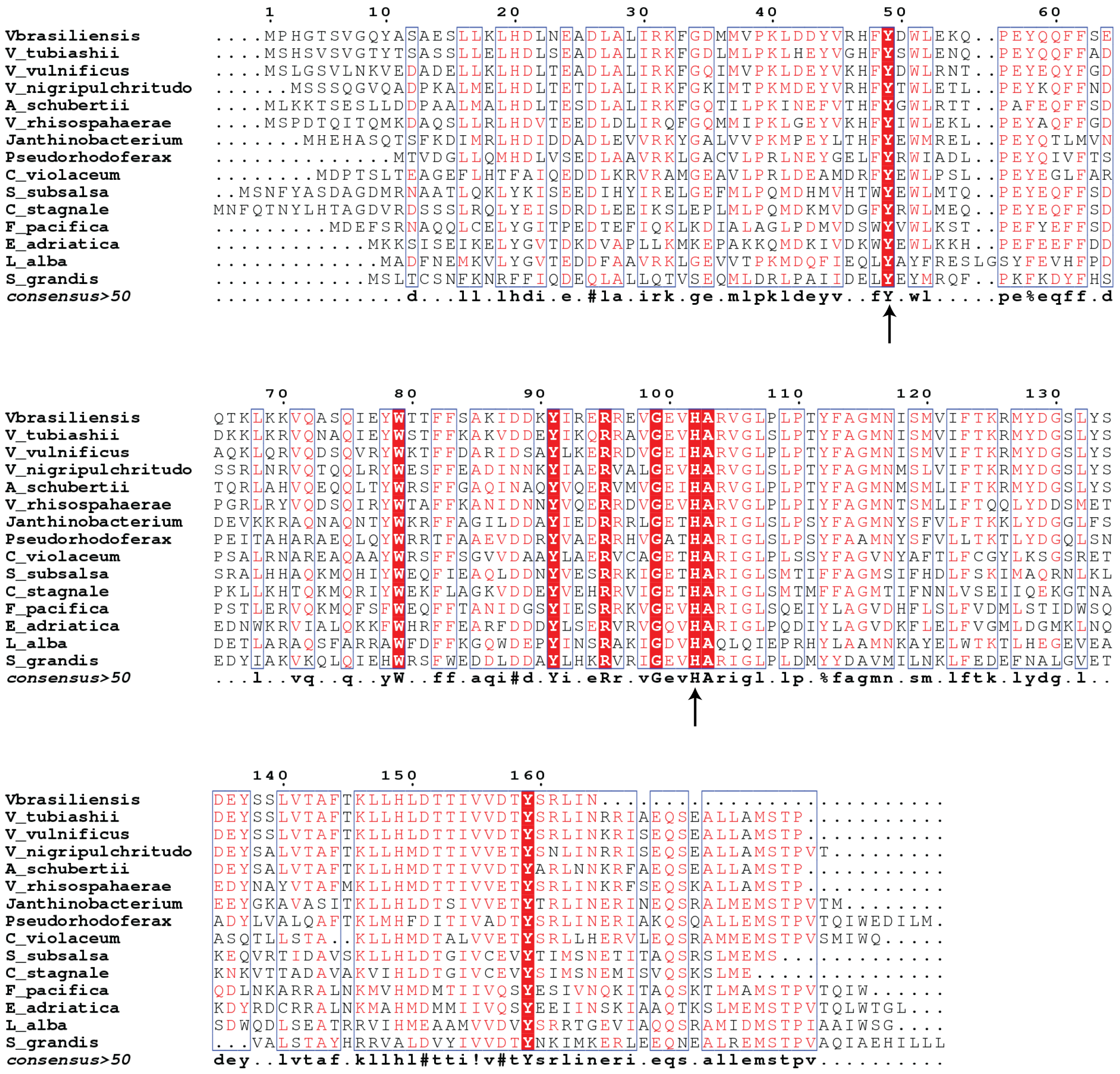

Supplementary Figure 14. Sequence alignment of sensor globin domains from putative RsbR proteins within the same phylogenetic branch as *Vb*RsbR. The conserved proximal histidine and distal tyrosine are highlighted.

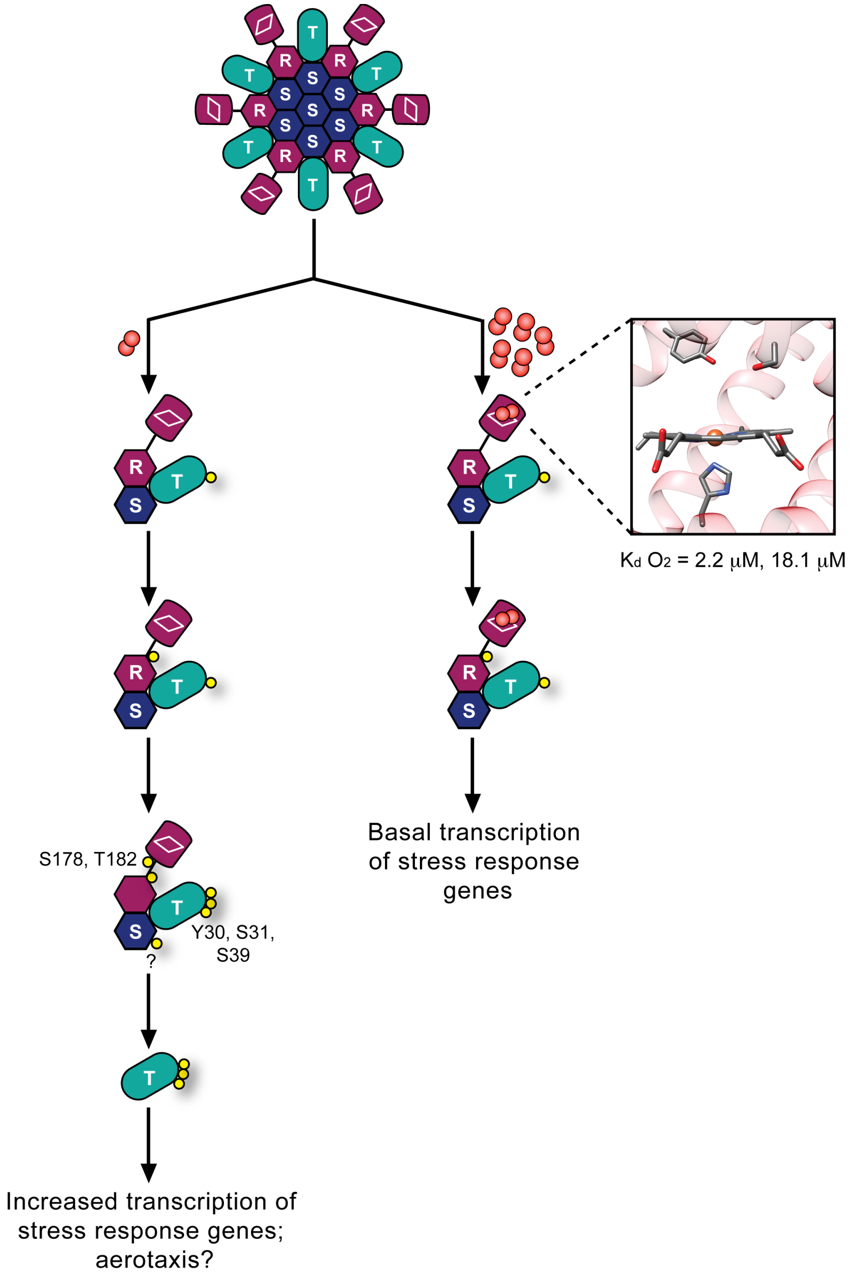

Supplementary Figure 15. Proposed O_2-sensing *V. brasiliensis* stressosome-regulated signaling pathway based on results described in this work. Under aerobic conditions (right), O_2 binds to the heme of RsbR (purple), and RsbT (turquoise) only phosphorylates RsbR, resulting in basal transcription level (top). However, when the bacteria enter low O_2 environments (left), RsbT is activated and leads to phosphorylation of RsbR at multiple sites; increasing levels of phosphorylated RsbR causes RsbS (blue) to also be phosphorylated by RsbT. Phosphorylated RsbS reduces the binding affinity of RsbT to the stressosome core, causing RsbT to activate downstream stress response gene transcription (bottom). Identified phosphorylation sites are listed.

Supplementary Table 1. Absorbance maxima of RsbR heme spectra.

Ligation/Oxidation State	Absorbance Maxima
Fe^{II}	432, 556 nm
Fe^{II}-O_2	417, 544, 581 nm
Fe^{II}-CO	422, 540, 569 nm
Fe^{II}-NO	419, 549, 569 nm
Fe^{III}	408, 534, 566 nm

Supplementary Table 2. Rates of phosphorylation of RsbR/S/T mixture in the presence of RsbR in various ligation/oxidation states. Rates and errors were obtained by fitting the data shown in Figure 3 and Supplementary Figure 7 to a single exponential fit.

RsbR Heme State	Phosphorylation of RsbT (min^{-1})	Phosphorylation of RsbR (min^{-1})	Phosphorylation of RsbS (min^{-1})
Fe^{II}-unliganded	0.007 ± 0.003	0.068 ± 0.005	0.032 ± 0.009
Fe^{II}-NO	0.022 ± 0.004	0.051 ± 0.008	0.014 ± 0.004
Fe^{II}-CO	0.026 ± 0.005	0.027 ± 0.004	0.010 ± 0.009
Fe^{II}-O_2	0.010 ± 0.001	0.016 ± 0.001	Not detected
Fe^{III}	0.005 ± 0.002	0.009 ± 0.002	Not detected

Supplementary Table 3. Phosphorylation sites identified in RsbR and RsbT using a mass spectrometry-based proteomics approach. Listed peptides were observed in both kinase reaction samples. The site of thiophosphorylation is highlighted in bold and underlined.

Protein	Phosphorylation Site	Identified Peptide
RsbR	S178	IAEQSEALLAM**S**TPVTMVWQDILMLPIVGIIDSK
RsbR	T182	IAEQSEALLAMSTPV**T**MVWQDILMLPIVGIIDSK
RsbT	Y30	IASETEAMSAVIAV**Y**SLGQNQALSATEVSEMSTSVSELAMNIVK
RsbT	S31	IASETEAMSAVIAVY**S**LGQNQALSATEVSEMSTSVSELAMNIVK
RsbT	S39	IASETEAMSAVIAVYSLGQNQAL**S**ATEVSEMSTSVSELAMNIVK

Supplementary Table 4. PCR Primers.

Name	Sequence 5'→ 3'
rsb-for	TTTGAATTCCATATGCCACATGGAACTTCGGTTG
rsb-rev	TAGCATTGACGCGATTTTGCGC
His-for	CATATGCACCACCACCACCACCACATGAAAATCGAAGAAGGTA AAC
His-rev	GAATTCTGACTGGAAGTACAGGTTCTCTGAA ATCCTTCCCTCG
rsbRpMAL-for	TTTCAGAATTCGGATCCTCTAGAATGCCACATGGAACTTCGGTTG
rsbRpMAL-rev	AAACGACGGCCAGTGCCAAGCTTTCACTCAGACAGCTGTTTTAAAG
rsbTpMAL-for	CGAGGGAAGGATTTTCAGAATTCATGCCCGACAGTGACTAC
rsbTpMAL-rev	AAACGACGGCCAGTGCCAAGCTTTTAGGATTTCCATTTAA
rsbSpGEX-for	TTCCGCGTGGATCCCCGGAATTCATGTCAAAGTCTATTTCA
rsbSpGEX-rev	CAGTCACGATGCGGCCGC TCGAGTCACTGTCGGGCACAAGC
rsbRTev-for	GATCGAGGGAAGGATTTCAGAGAACCTGTACTTCCAGGGCGAATTCGGAT CCTCTAG
rsbRTev-rev	CTAGAGGATCCGAATTCGCCCTGGAAGTACAGGTTCTCTGAAATCCTTCCC TCGATC
rsbTTev-for	GATCGAGGGAAGGATTTCAGAGAACCTGTACTTCCAGGGCGAATTCATGC CCGACAG
rsbTTev-rev	CTGTCGGGCATGAATTCGCCCTGGAAGTACAGGTTCTCTGAAATCCTTCCC TCGATC
rsbSTev-for	GATCGAGGGAAGGATTTCAGAGAACCTGTACTTCCAGGGCGAATTCATGT CAAAGTC
rsbSTev-rev	GACTTTGACATGAATTCGCCCTGGAAGTACAGGTTCTCTGAAATCCTTCCC TCGATC

Supplementary References

1 Dereeper, A. *et al.* Phylogeny.fr: robust phylogenetic analysis for the non-specialist. *Nucleic acids research* **36**, W465-469, doi:10.1093/nar/gkn180 (2008).
